# Magnitude and factors associated with iron supplementation among pregnant women in Southern and Eastern Regions of Ethiopia: Further Analysis of mini demographic and health survey 2019

**DOI:** 10.1186/s40795-022-00562-3

**Published:** 2022-07-18

**Authors:** Girma Teferi Mengistu, Bizunesh Kefale Mengistu, Tolesa Gemeda Gudeta, Ayana Benti Terefe, Fedhesa Mamo Habtewold, Mebratu Demissie Senbeta, Seboka Abebe Sori, Hirut Dinku Jiru

**Affiliations:** 1 Department of Nursing, College of Medicine and Health Science, Wolkite University, Wolkite, Ethiopia; 2 Department of Statistics, College of Natural and Computational Science, Ambo University, Ambo, Ethiopia; 3 Department of Midwifery, Jimma University Medical Center, Jimma, Ethiopia; 4 Department of Midwifery, College of Medicine and Health Science, Wolkite University, Wolkite, Ethiopia

**Keywords:** Pregnant women, Iron, Iron supplementation, Sothern and eastern regions, Ethiopia

## Abstract

**Background:**

Anemia is a global public health problem that affects pregnant women. The most common cause of anemia is iron deficiency which is extremely common in developing countries. World health organization reported that 36.5% of pregnant women are anemic globally. In Ethiopia, 27.08% of women of the reproductive age group are anemic. Therefore, this study aimed to identify the magnitude and factors associated with iron supplementation during pregnancy in the southern and eastern regions of Ethiopia.

**Methods:**

The data used in this analysis were extracted from Mini Demographic and Health Survey 2019. The survey was conducted in 9 regional states and two city administrations. The data used in the analysis were extracted from individual women datasets, and 1780 study participants were included in this study. The logistic regression analysis including bivariate and multivariable logistic regression at a 95% confidence interval and a *p*-value less than 0.05 was used.

**Result:**

The finding of the study shows that iron supplementation during pregnancy in Southern and Eastern parts of Ethiopia was 50.06%. Among those who received iron, only about 20% took it for 90 days and more during their pregnancy. Iron supplementation among the pregnant women was affected by secondary education [AOR = 2.20, 95%CI (1.325, 3.638)], residing in urban [AOR = 1.75, 95%CI (1.192, 2.574)], having media at home [AOR = 1.41, 95%CI (1.022, 1.946)], having antenatal care follow up [AOR = 9.27, 95%CI (4.727, 18.169)], having 4 and more ANC follow up [AOR = 2.01, 95%CI (1.468,2.760], having antenatal care follow up at government health institutions [AOR = 3.40, 95%CI (1.934, 5.982)], and giving birth at governmental health institutions [AOR = 1.70, 95%CI (1.236, 2.336)].

**Conclusion:**

Only one in two pregnant women was supplemented with iron during their recent pregnancy. The supplementation was affected by women's education, place of residence, presence of media at home, antenatal care follow-up, the number of antenatal care follow up, antenatal care follows up at governmental health institutions, and giving birth at the governmental health institution. The availability and accessibility of maternal care services and their functionality in providing maternal care services improve the supplementation.

## Introduction

Anemia in pregnancy is defined as a hemoglobin concentration of 11 gm/dl or less in the peripheral blood, which is diagnosed by examining characteristics of red blood cell changes in peripheral blood smear [1, 2]. Anemia occurs when there are not enough red blood cells or hemoglobin in the blood. Hemoglobin, which is the part of the red blood cells that carries oxygen, is made of iron [3]. Pregnant women are vulnerable to iron deficiency due to more iron being needed primarily to supply the growing fetus and placenta, which increases the maternal red cell mass [4]. The problem is that if pregnant women become deficient in these nutrients, they are not able to provide them an adequate amount for their baby, which can lead to anemia and increases the risk of complications [5].

Iron supplementation is the best way of reducing iron deficiency anemia [6]. Pregnant mothers are advised to take iron supplementation for 180 days during pregnancy [7]. In addition to iron supplementation, pregnant women should be advised to eat foods high in iron and prevent intestinal worms [8].

Anemia is a global public health issue that affects pregnant mothers [9]. Anemia during pregnancy results in a variety of life-threatening complications and poor pregnancy outcomes [3]. Iron deficiency is the most common dietary deficiency globally with negative maternal and perinatal outcomes [10]. It is extremely common in developing countries as a global epidemic where many women go through the whole pregnancy without reaching the required minimal iron intake [11]. Maternal anemia during the first or second trimester of pregnancy increases the risk of prematurity and being underweight at birth [12]. Due to increased iron requirements during pregnancy, iron deficiency can cause maternal anemia and reduce the iron reserves of the newborn [13]. Severe anemia can significantly increase the risk of maternal mortality and negatively affects fetal development [14]. But early initiation and use of a greater number of iron supplements reduce the risk of early neonatal complications [15].

According to World Health Organization estimates in 2019, the prevalence of anemia among pregnant women was 36.5% globally [16]. Similarly, the finding of a study conducted among 10 east African countries shows that the prevalence of anemia among reproductive-aged women was 34.85%. This study identified that the prevalence ranges from 19.23% in Rwanda to 53.98% in Mozambique [17]. The report from further analysis of Ethiopia demographic and health survey (EDHS) 2016 shows that 27.08% of reproductive-aged women were anemic in Ethiopia [18]. It is highest in the Somali and Afar regions with 68.3% and 47.2% respectively [19].

Even though iron supplementation is used to prevent iron deficiency anemia in pregnancy, the report of EDHS 2016 shows that iron supplementation during pregnancy was 42% in Ethiopia [20]. The supplementation was showing improvement as evidenced by the report of Ethiopia Mini Demographic and Health Survey (EMDHS) 2019. This report identified that iron supplementation during pregnancy was 60% [8].

Iron supplementation during pregnancy can reduce maternal anemia at term by 70% by increasing their hemoglobin concentration [5]. However, it is affected by the women’s level of adherence to the supplementation. The finding from the systematic review done on adherence to iron and folic acid supplementation in Ethiopia shows that nationally only 46.15% of the women adhered during pregnancy [21].

There are plenty of studies that have been done to identify factors associated with compliance to iron supplementation during pregnancy [22–26]. These studies do not address iron factors associated with supplementation. There was one study reporting determinants of iron supplementation nationally which reported a low level of iron supplementation in southern and eastern regions of the country [27]. Despite the low level of iron supplementation in these regions, there was a lack of evidence reporting factors affecting iron supplementation in the regions during pregnancy.

Therefore, this study aimed to identify the magnitude and factors associated with iron supplementation in the Southern and Eastern Regions of Ethiopia based on the Ethiopia mini demographic and health survey 2019. The finding of the study will help in improving maternal healthcare services.

## Methodology

### Data source

The data used in this analysis were obtained from Ethiopia Mini Demographic and Health Survey (DHS) 2019. Mini DHS 2019 is a national-level survey that gathers information about children, women, and men and analyses and interprets the findings. The survey was done nationally among 9 Regional States and two City Administrations found in the country. The data collection period for mini DHS 2019 was from March and June 2019 [8].

### Sampling procedure

This survey used two stages of sampling frame to collect the data at the national level. During the first stage, all census enumeration areas (EAs) created for the 2019 Population and Housing Census were used. And 305 EAs were selected with probability proportional to EA size and with independent selection in each sampling stratum. The second stage of selection used a fixed number of 30 households per cluster with an equal probability of systematic selection from the newly created household list. All women of reproductive age, fulfilling the selection criteria were eligible for the survey and selected for interviews [8].

For the current study, we used the 4 Regional States and one City Administration found in the Eastern and Southern parts of the country. The regions included in this analysis were Southern Nation Nationalities and Peoples Region, Harari Region, Dire Dawa City Administration, Somali Region, and Afar Region. This study used data from individual women datasets and a total weighted sample of 1780 women of reproductive age was included in the study. This sample was extracted from 8885 women interviewed during the survey. Among the respondents who participated in the survey, the data of 3964 respondents were recorded for the outcome variable at the national level. Then, since this study was based on the data from southern and eastern regions of the country, we extracted 1780 samples based on the regions included in the analysis.

## Variables

### Dependent

The dependent variable for the study was factors associated with iron supplementation during pregnancy. The variable was derived from the individual women dataset which has maternal-related information. The outcome variable was categorized and coded as ‘Yes’ (1) and ‘No’ (0).

### Independent

The independent variables included in the current study were categorized under two categories. The first category was sociodemographic variables such as age (‘ ≤ 24’, ‘25–29’, ‘30–34, ≥ 35’), educational attainment (‘no education’, ‘primary education, ‘secondary education and above’), media in a home (‘yes’, ‘no’, whether television or radio is present in the home), place of residence (‘urban’, ‘rural’), Region (‘Afar’, ‘Somali’, ‘SNNPR’, ‘Harari’, ‘Dire Dawa’), marital status (‘unmarried/in a relationship’, ‘married’, ‘widowed/divorced/separated), wealth index (‘poor’, ‘middle’, ‘rich’). The second category was obstetric characteristics: pregnant during the survey (‘yes’, ‘no’), has ANC follow up in the last 5 years preceding the survey (‘yes’, ‘no’), number of ANC follow ups for the last recent pregnancy in the last 5 years preceding the survey (‘less than’, ‘4 more), place of ANC follow up (‘home’, ‘governmental heath institutions’, ‘private health facilities’), age of respondent at first birth (‘ ≤ 14’, ‘15–19’, ‘20–24’, ‘ ≥ 25’), place of delivery (‘home’, ‘governmental heath institutions’, ‘private health institutions’), delivery by cesarean section (‘yes’, ‘no’), number of births in last 5 years (‘1–2’, ‘ ≥ 3’), number of births in last 3 years (‘no births’, ‘1 birth’, ‘2–3 births’), and number of living children (‘no children’, ‘1–2’, ‘3–4’, ‘ ≥ 5’).

### Operational definition

Iron supplementation during pregnancy: If the respondents were supplemented or told to buy iron tablets or syrup from the pharmacy during their antenatal care follow-up.

### Data analysis

The extracted data were analyzed using computer software, SPSS version 23. The analysis of the data included both descriptive and inferential statistics. The descriptive analysis was used to analyze the frequency distribution of the data. The descriptive analysis included a presentation of data using frequency tables and figures with their description. The logistic regression analyses were used to check for the relation of the independent variable with the dependent variable, iron supplementation during pregnancy. Multicollinearity was checked for independent variables used in multivariable logistic regression analysis using the variation inflation factor. The logistic regression included bivariate and multivariable logistic regression analysis at a 95% confidence interval and *p*-value less than 0.05. In bivariate logistic regression, each variable was checked with the outcome variable. Those variables with a *p*-value of less than 0.25 were used for multivariable logistic regression analysis. Then variables with a *p*-value of less than 0.05 in multivariable logistic regression were declared as statistically significant with iron supplementation during pregnancy. The output of the logistic regression analysis was presented with an expression of odd ratios. The crude odds ratio was used for bivariate logistic regression while the adjusted odds ratio was used for multivariable logistic regression.

#### Ethical consideration

The authors received permission to use the data from DHS, and the data set is available online at http://www.dhsprogram.com/data/available-datasets.cfm.

## Results

### Sociodemographic characteristics

There were 1780 samples included in the analysis of the study. Among the study participants, 576 (32.4%) were found in the age category of 25–29. Regarding the educational status of the women 1065 (59.8%) of them were not educated, followed by primary education 503 (28.3%). Nearly two-thirds, 1128 (63.4%) of the respondents reported that they have media in their homes. The finding of the study also indicated that almost three fourth 1313 (73.8%) of them were from rural areas. Regarding marital status, almost all 1671 (93.9%) of the respondents were married. Almost one-half, 942 (52.9%) of the respondents were from poor families (Table 1).

**Table 1:** Sociodemographic characteristics of reproductive age women, southern and eastern regions of Ethiopia

Variables	Characteristics	Frequency	Percent
**Age of respondent**	$\le$ 24	464	26.1
	25–29	576	32.4
	30–34	360	20.2
	$\ge$ 35	380	21.3
**Educational attainment**	No education	1065	59.8
	Primary education	503	28.3
	Secondary and above	212	11.9
**Media in home**	Yes	1128	63.4
	No	652	36.6
**Place of residence**	Urban	467	26.2
	Rural	1313	73.8
**Region**	Afar	387	21.7
	Somali	340	19.1
	SNNPR	466	26.2
	Harari	307	17.2
	Dire Dawa	280	15.7
**Marital status**	unmarried/in relationship	15	0.8
	Married	1671	93.9
	widowed/divorced/separated	94	5.3
**Wealth index**	Poor	942	52.9
	Middle	177	9.9
	Rich	661	37.1

### Obstetrics characteristics

**Fig. 1:**
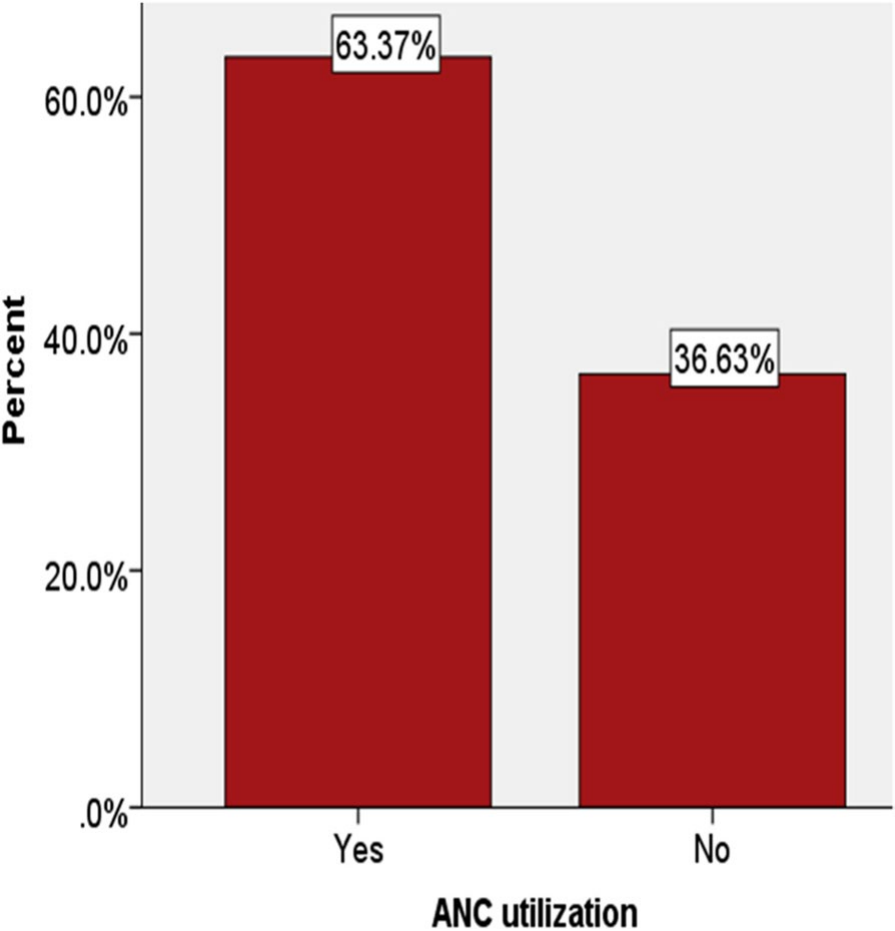
Antenatal care service utilization in Southern and Eastern Regions of Ethiopia

**Fig. 2:**
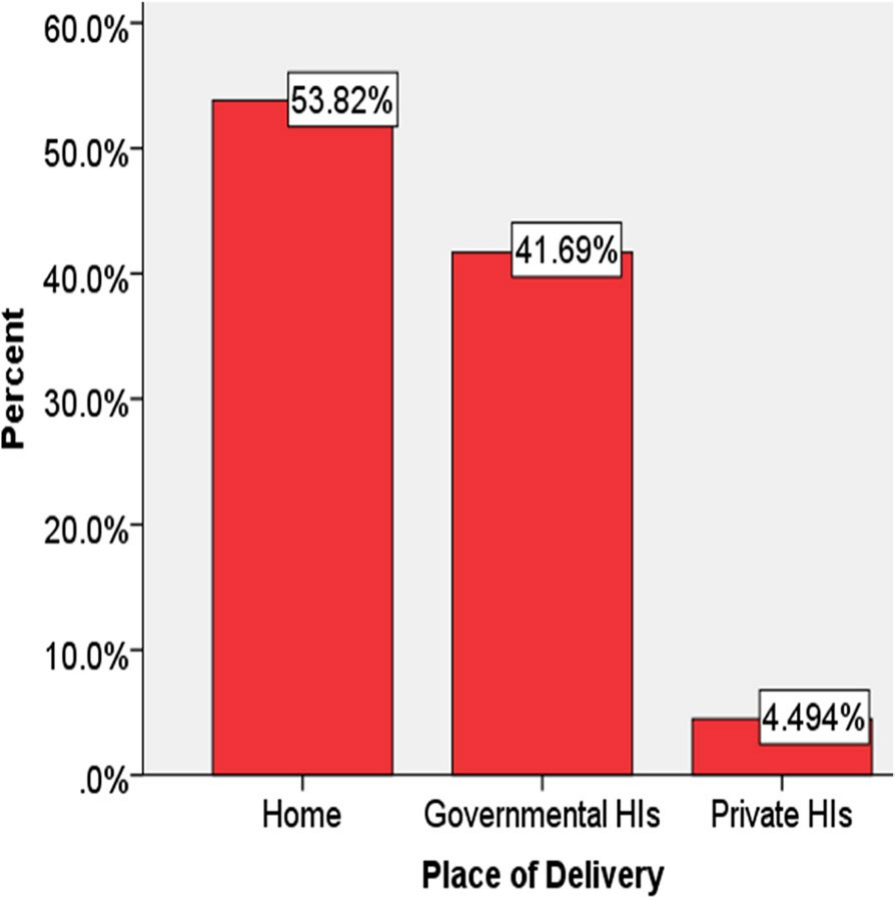
Distribution of place of delivery in Southern and Eastern Regions of Ethiopia

According to the current study, ANC service utilization was 1128 (63.4%), where only 575 (32.3%) of them had completed 4 visits (Fig. 1). Among those who had ANC follow up 981 (55.1%) of them were at governmental health institutions. Regarding the age of respondents at first birth, about half 852 (47.8%) of them gave birth between the age category of 15–19, while 258 (14.5%) of the gave birth before the age of 14 years old. More than one-half, 958 (53.8%) of the mothers gave birth at home (Fig. 2) as evidenced by the finding of the study (Table 2).

**Table 2:** Obstetrics characteristics of reproductive age women, southern and eastern regions of Ethiopia

Variables	Characteristics	Frequency	Percent
Pregnant during survey	No	1516	85.2
	Yes	264	14.8
ANC follow up	No	652	36.6
	Yes	1128	63.4
Number of ANC follow up	Less than 4	1205	67.7
	4 and more	575	32.3
ANC follows up at home	No	1765	99.2
	Yes	15	.8
ANC follows up at Government His	No	799	44.9
	Yes	981	55.1
ANC follows up at Private/NGO His	No	1630	91.6
	Yes	150	8.4
Age of respondent at first birth	$\le$ 14	258	14.5
	15–19	851	47.8
	20–24	521	29.3
	$\ge$ 25 +	150	8.4
Place of delivery	Home	958	53.8
	Governmental health institution	742	41.7
	Private health facility	80	4.5
Delivery by cesarean section	No	1662	93.4
	Yes	118	6.6
Number of births in last 5 years	1–2	1622	91.1
	$\ge$ 3	158	8.9
Number of births in last 3 years	No birth	394	22.1
	1 birth	1143	64.2
	2–3 births	243	13.7
Number of living children	no child	22	1.2
	1–2	681	38.3
	3–4	494	27.8
	$\ge$ 5	583	32.8

### Iron supplementation

**Fig. 3:**
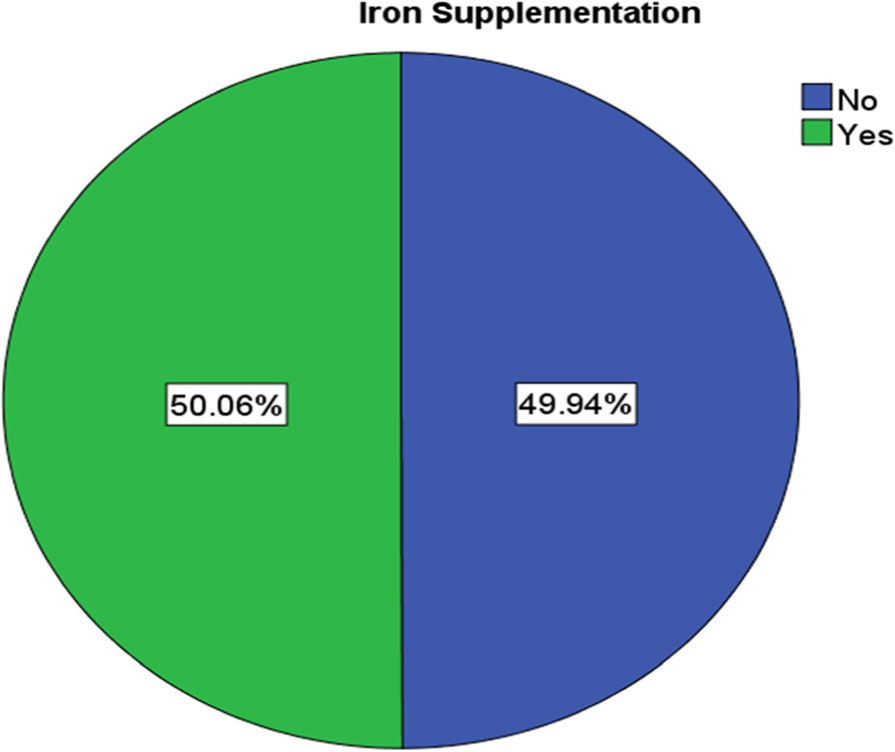
Iron supplementation in Southern and Eastern Regions of Ethiopia

**Fig. 4:**
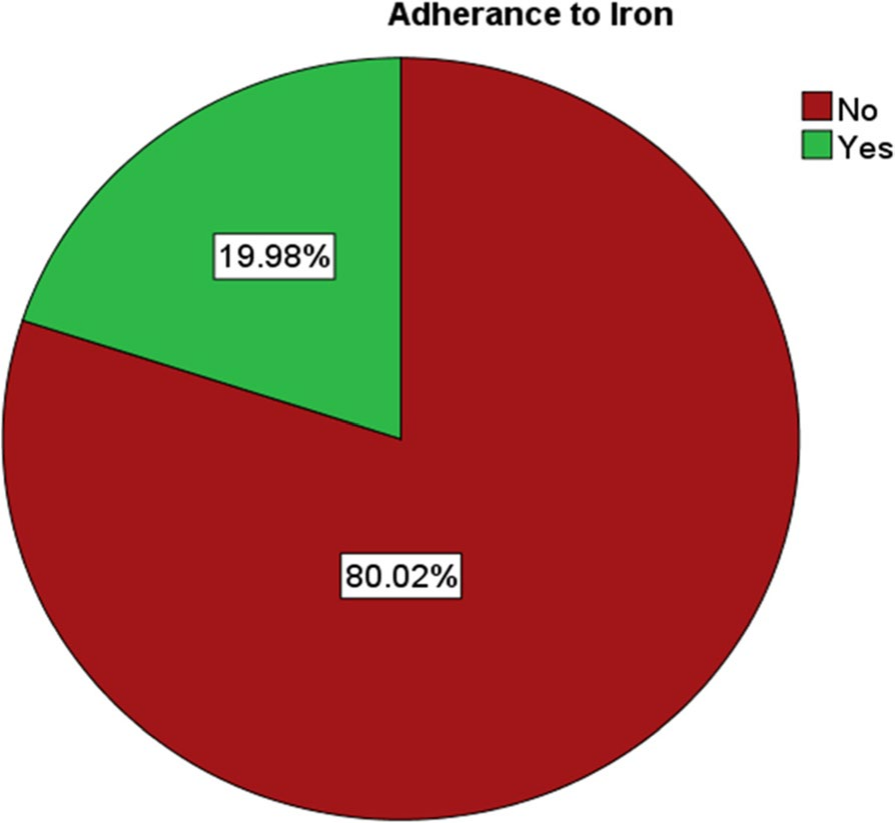
Adherence of mothers towards iron in southern and eastern regions of Ethiopia

The finding from the analysis of the data shows that iron supplementation during pregnancy in Southern and Eastern parts of Ethiopia was 50.06% (Fig. 3). Among those who received iron only about 20% took it for 90 days and more during their pregnancy (Fig. 4).

### Factors associated with iron supplementation during pregnancy

The logistic regression analysis was conducted to identify the relation between the dependent and independent variables. We conducted bivariate and multivariable logistic regression analysis at 95%CI and declared a significant association with a *p*-value < 5%.

The bivariate analysis indicated that age of respondents, educational status, residence, region, media at home, ANC follow up, place of ANC follow up, number of ANC follow up, place of delivery, number of births in the last 5 years, the number of births in the last 3 years, the number of living children were statistically significant with the outcome variable (iron supplementation). After controlling the confounding factors in Multivariable analysis, educational status, media in a home, residence, region, ANC follow up, number of ANC follow up, ANC visit at governmental health institutions, and place of delivery was statistically significant.

Women with secondary education and above had 2.20 [AOR = 2.20, 95%CI (1.325, 3.638)] times higher odds of iron supplementation during pregnancy than women with no education. Mothers who live in urban areas had 1.75 [AOR = 1.75, 95%CI (1.192, 2.574)] times higher odds of iron supplementation during pregnancy than mothers living in rural areas. Women who had a television or radio in their home had 1.41 [AOR = 1.41, 95%CI (1.022, 1.946)] times higher odds of iron supplementation than mothers who had no television or radio in their home. Mothers who had ANC follow-up had 9.27 [AOR = 9.27, 95%CI (4.727, 18.169)] times higher odds of iron supplementation than mothers who had no ANC follow-up. Mothers who have four and more ANC follow-ups had 2.01 [AOR = 2.01, 95%CI (1.468,2.760] times higher odds of iron supplementation than mothers who had less than 4 ANC follow up. Mothers who had ANC follow-up at government health institutions had 3.40 [AOR = 3.40, 95%CI (1.934, 5.982)] times higher odds of iron supplementation than mothers who had no ANC follow-up at governmental health institutions. Women who gave birth at governmental health institutions had 1.70 [AOR = 1.70, 95%CI (1.236, 2.336)] times higher odds of iron supplementation than mothers who gave birth at home (Table 3).

**Table 3:** Determinants of iron supplementation for pregnant women, Southern and Eastern Regions of Ethiopia

Variable	Characteristics	Received Iron		COR95%CI	AOR95%CI
		Yes	No		
Age of respondent	$\le$ 24	240(51.7%)	224(48.3%)	1.52(1.158, 2.001)**	
	25–29	311(54.0%)	265(46.0%)	1.66(1.283, 2.166)***	
	30–34	183(50.8%)	177(49.2%)	1.46(1.098, 1.964)*	
	$\ge$ 35	157(41.3%)	223(58.7%)	1	
Educational attainment	No education	409(38.4%)	656(61.6%)	1	1
	Primary education	304(60.4%)	199(39.6%)	2.45(1.972, 3.045)***	1.21(0.883,1.648)
	Secondary & above	178(84.0%)	34(16.0%)	8.39(5.702, 12.366)***	1.19(0.872, 1.630)**
Residence	Urban	331(70.9%)	136(29.1%)	3.27(2.606, 4.109)***	2.20 (1.325,3.638)**
	Rural	560(42.7%)	753(57.3%)	1	1
Media in home	No	421(64.6%)	231(35.4%)	1	1
	Yes	470(41.7%)	658(58.3%)	0.39(0.321, 0.478)***	1.41(1.022,1.946)*
Region	SNNPR	190(49.1%)	197(50.9%)	1	1
	Somali	58(17.1%)	282(82.9%)	0.87(0.670, 1.149)	0.39(0.238, 0.626)***
	Afar	244(52.4%)	222(47.6%)	0.18(0.134, 0.262)***	0.68(0.460,1.004)
	Harari	207(67.4%)	100(32.6%)	1.88(1.395, 2.542)***	1.17(0.740, 1.867)
	Dire Dawa	192(68.6%)	88(31.4%)	1.98(1.455, 2.709)***	0.93(0.582,1.479)
ANC follow up	No	858(76.1%)	270(23.9%)	1	1
	Yes	33(5.1%)	619(94.9%)	59.60(40.930, 86.808)***	9.27(4.727, 18.169)***
Number of ANC follow up	Less than four	411(34.1%)	794(65.9%)	1	1
	Four and more	480(83.5%)	95(16.5%)	9.76(7.600, 12.537)***	2.01(1.468,2.760)***
ANC follow up: Governmental HIs	No	131(16.4%)	668(83.6%)	1	1
	Yes	760(77.5%)	221(22.5%)	17.53(13.797, 22.288)***	3.40(1.934, 5.982)***
Place of delivery	Home	280(29.2%)	678(70.8%)	1	1
	Governmental HIs	563(75.9%)	179(24.1%)	7.61(6.122, 9.474)***	1.70(1.235, 2.333)**
	Private/NGO HIs	48(60.0%)	32(40.0%)	3.63(2.274, 5.803)***	0.97(0.483,1.949)
Number of births in last 5 years	1–2	832(51.3%)	790(48.7%)	1.76(1.262, 2.475)**	
	$\ge$ 3	59(37.3%)	99(62.7%)	1	
Number of births in last 3 years	no births	198(50.3%)	196(49.7%)	1.32(0.963, 1.831)	
	1 birth	588(51.4%)	555(48.6%)	1.39(1.053, 1.841)*	
	2–3 births	105(43.2%)	138(56.8%)	1	
Number of living children	no children	8(36.4%)	14(63.6%)	1	
	1–2	405(59.5%)	276(40.5%)	2.56(1.063, 6.204)*	
	3–4	238(48.2%)	256(51.8%)	1.62(.671, 3.948)	
	$\ge$ 5	240(41.2%)	343(58.8%)	1.22(.506, 2.964)	

^*^p < 0.05, **p < 0.01, ***p < 0.001

## Discussion

The finding of the current study identified that the magnitude of iron supplementation during pregnancy was 50.06% in the Southern and Eastern Regions of the country. This finding was lower than studies conducted in Germany, 65.2% [28], Khartoum Sudan, 92.1% [29], and Ethiopia, 60.87% [27]. The inconsistency could be related to geographical and socio-economical differences. Regions with poor socioeconomic status and remote areas have barriers to health care service [30].

On the other hand, this finding was higher than studies reported from Tanzania, 22.3% [31], and eight rural districts of Ethiopia, 35.4% [22]. These two studies were done before 14 years and 8 years respectively. The health delivery system is improving with time frame even though it is not satisfactory in some of the regions.

Among those who received iron supplementation, only 20% of them took it for 90 days and above during their pregnancy which is supported by another study [27]. On the other hand, the supplementation has increased when compared to the EDHS 2016 report where 42% of the mother were supplemented with iron during pregnancy [20]. Regionally the supplementation was lowest in the Somali Region where less than one-fifth of pregnant mothers received the supplements. Somali and Afar regions had a higher prevalence of anemia, 68.3% and 47.2% respectively [19]. But women of reproductive age have perceived barriers to healthcare service access, 69.9% [30].

The percentage of pregnant women who received antenatal care service was 63.4%, and only 32.3% of them has 4 or more ANC visits. This finding is similar to the study reported five years back, 62% of pregnant women received ANC service as evidenced by the report of EDHS 2016 [20]. On the other hand, the EMDHS 2019 report shows that the utilization of ANC service was 74.4%, which is higher than the current finding [27]. The difference between the studies indicates that the health delivery system of these Regions is fragile even affecting the report at the country level [27].

In those Regions, institutional delivery was less than half, where only 46.18% of the pregnant mothers gave birth at governmental or private health institutions. More than half of the mothers gave birth at home without the assistance of health professionals. The mothers gave birth at home due to a lack of awareness about maternal care benefits [32]. Institutional delivery was slightly higher at the national level, 52.5% as reported by EMDHS 2019. Additionally, EDHS 2016 report shows that 48% of pregnant women gave birth, five years preceding the survey [20]. The service provision in these Regions indicates that it is five years backward from the other parts of the country. The difference in the report could be related to the geographical location and access to facilities such availability of health institutions and roads. The finding of the study identified that 14.5% of mothers gave birth before the age of 14. Delivery before the age of 14 years old can result from early marriage caused by poor socioeconomic status, lack of education at the community level, and cultural or religious related [33].

Iron supplementation was determined by the educational status of mothers, having media at home, place of residence, Region, ANC follow-up, number of ANC follow up, and place of delivery.

Educational status has a positive effect on iron supplementations [34]. Mothers with secondary and above education had 2.20 higher odds of iron supplementation during pregnancy than mothers with no education. This could be related to exposure to information about the purpose of iron during pregnancy. Mothers who live in urban had 1.75 higher odds of iron supplementation during pregnancy than mothers from rural areas. This could have resulted from access to health facilities and information related to iron supplementation. Those mothers who had a radio or television in their home had 1.41 higher odds of iron supplementation during pregnancy than those who had no radio or television where they could acquire information.

Mothers living in the Somali region had lower odds of iron supplementation during pregnancy than mothers living in the SNNP region. This finding reflects the health delivery system of the region is not coordinated. On the other hand, mothers living in Afar and Harari had higher odds of receiving iron supplementations than mothers living in SNNPR. The population living in Afar and Harari regions is lower than SNNPR, and they could afford the health care delivery system.

Mothers who had ANC follow-ups had 9.27 higher odds of iron supplementation during pregnancy than those who had no ANC follow up. Mothers who had 4 or more ANC follow-ups had 2.01 higher odds of iron supplementation during pregnancy than those who had less than 4 ANC follow up. It is supported by a study from Sudan [29]. During ANC visits, iron supplementation is provided as one component of ANC service. Those mothers who had ANC follow-up at governmental health institutions had 3.40 higher odds of iron supplementation during pregnancy than others which is supported by another study [35]. At governmental health institutions, iron is freely supplemented. Mothers who gave birth at governmental health institutions had 1.70 higher odds of iron supplementation during pregnancy than those who gave birth at home. Mothers are counseled after delivery about their health and nutrition. Depending on their health status, they could be informed about the prevention of anemia through iron supplementation.

This study was done based on secondary data which was extracted from EMDHS 2019, and four regions and one city administration found in the southern and eastern parts of the country were studied. In this study, variables were excluded from the analysis because of unregistered data or missing values. Among the variables, hemoglobin and level of anemia were not included since no data was entered for the variables.

## Conclusion

Even though iron supplementation during pregnancy prevents iron deficiency anemia, the current study identified that only one in two were supplemented. It was also evidenced that among those who have supplemented, only one-fifth of them received for 90 and more days. Iron supplementation during pregnancy was affected by maternal education, place of residence, having media at home, having antenatal care follow-up, having antenatal care follow-up at a governmental health institution, and place of delivery of the last recent baby. Availability and functionality of maternal care services will facilitate iron supplementation during pregnancy. Access to a health facility in nearby areas of their residential area helps in enabling the mothers to receive the service.

## Data Availability

The dataset used in this study is available on the hand of the corresponding author upon reasonable request.
